# Deep learning-based multimodality classification of chronic mild traumatic brain injury using resting-state functional MRI and PET imaging

**DOI:** 10.3389/fnins.2023.1333725

**Published:** 2024-01-19

**Authors:** Faezeh Vedaei, Najmeh Mashhadi, Mahdi Alizadeh, George Zabrecky, Daniel Monti, Nancy Wintering, Emily Navarreto, Chloe Hriso, Andrew B. Newberg, Feroze B. Mohamed

**Affiliations:** ^1^Department of Radiology, Jefferson Integrated Magnetic Resonance Imaging Center, Thomas Jefferson University, Philadelphia, PA, United States; ^2^Department of Computer Science and Engineering, University of California, Santa Cruz, Santa Cruz, CA, United States; ^3^Department of Integrative Medicine and Nutritional Sciences, Marcus Institute of Integrative, Health, Thomas Jefferson University, Philadelphia, PA, United States

**Keywords:** deep learning, artificial neural network, resting-state functional MRI, positron emission tomography, mild traumatic brain injury, classification

## Abstract

Mild traumatic brain injury (mTBI) is a public health concern. The present study aimed to develop an automatic classifier to distinguish between patients with chronic mTBI (*n* = 83) and healthy controls (HCs) (*n* = 40). Resting-state functional MRI (rs-fMRI) and positron emission tomography (PET) imaging were acquired from the subjects. We proposed a novel deep-learning-based framework, including an autoencoder (AE), to extract high-level latent and rectified linear unit (ReLU) and sigmoid activation functions. Single and multimodality algorithms integrating multiple rs-fMRI metrics and PET data were developed. We hypothesized that combining different imaging modalities provides complementary information and improves classification performance. Additionally, a novel data interpretation approach was utilized to identify top-performing features learned by the AEs. Our method delivered a classification accuracy within the range of 79–91.67% for single neuroimaging modalities. However, the performance of classification improved to 95.83%, thereby employing the multimodality model. The models have identified several brain regions located in the default mode network, sensorimotor network, visual cortex, cerebellum, and limbic system as the most discriminative features. We suggest that this approach could be extended to the objective biomarkers predicting mTBI in clinical settings.

## Introduction

Mild traumatic brain injury (mTBI) is a growing public health concern worldwide that can result in a broad spectrum of short-term and long-term symptoms, including a decline in cognitive functioning and mobility (e.g., poor concentration, slowed thinking, memory loss, sleep disruption, fatigue, and irritability). Annually, mTBI accounts for more than 2 million deaths in the United States and over 10 million hospitalizations across the world (Vergara et al., 2017). Several incidents may cause TBI, such as falls, vehicle accidents, athletic collisions, blast-related trauma, and abuse or assault (Vedaei et al., 2021). A great deal of effort has been made to develop biomarkers of mTBI, including the application of advanced neuroimaging techniques to integrate large-scale high-dimensional multimodal neuroimaging data. Conventional computed tomography (CT) and magnetic resonance imaging (MRI) fail to detect any indication of mTBI in most of the cases, while cognitive dysfunction after mTBI has been known to be related to brain function disruption. Resting-state functional MRI (rs-fMRI) and positron emission tomography (PET) have been used to characterize intrinsic brain function, connectivity, and glucose metabolism, thus non-invasively aiding in the diagnosis of neurological and psychiatric disorders (Byrnes et al., 2014; O’Neill et al., 2017). A critical challenge in the analysis of rs-fMRI data owing to their high dimensionality is to extract meaningful patterns and derive insights from the data. Several methods have been used to analyze rs-fMRI data, such as functional segregation and integration analysis. Functional segregation focuses on the local function of specific regions incorporating spontaneous neuronal activity measured by rs-fMRI data including the fractional amplitude of low-frequency fluctuation (fALFF) and regional homogeneity (ReHo), while functional integration focuses on rs-fMRI functional connectivity (FC) between different brain regions considering the brain as an integrated network, which contains degree centrality (DC), voxel-mirrored homotopic connectivity (VMHC), and FC strength (FCS) (Tononi et al., 1994; Pang et al., 2021).

Taking into account that mTBI symptoms are not detected by conventional neuroimaging approaches, several studies have explored brain function and metabolism using rs-fMRI and PET imaging. For instance, a prior study investigated fALFF and FC among patients with mTBI and found decreased fALFF in the frontal, temporal, and occipital lobes in mTBI patients compared to healthy controls (HCs). Additionally, using seed-based FC by locating the seed in the thalamic area, these studies reported higher FC with the frontal, parietal, and occipital lobes as well as decreased FC with the temporal areas in mTBI patients compared to HCs (Zhou et al., 2014). Another study found increased ReHo in the superior frontal and middle occipital regions as well as decreased ReHo in the inferior frontal, medial frontal, superior temporal, parahippocampus, supramarginal, and supplementary motor areas in mTBI patients compared to HCs (Liu et al., 2018). Using PET, a previous study on veterans with exposure to blast and diagnosed with mTBI compared the cerebral glucose metabolism between veterans with exposure to blast and those with non-blast exposure. Using the voxel-wise whole-brain analysis, the authors of the abovementioned study showed glucose metabolism reduction in the parietal cortex, left somatosensory, and right visual cortex in the blast-mTBI veterans compared to the non-blast veterans (Petrie et al., 2014). Despite various applications of neuroimaging modalities, the consensus is far from being reached using different measurements, highlighting the need for a comprehensive study using multimodality metrics in mTBI patients. Specifically, previous literature has suggested that different metrics of rs-fMRI and PET may be complementary to each other in representing brain function alteration from different perspectives, bringing about more worthy information (Zhou et al., 2019; Pang et al., 2021).

Previous works relied typically on mass univariate analysis (group-level analysis) and reported group-level differences in specific brain areas, while they do not allow statistical inferences at the level of individuals. Thus, mass univariate analysis (group-level analysis) and reported group-level differences in specific brain areas may contribute to the limited translational impact of neuroimaging findings in clinical practice. Given the limitations of mass univariate techniques to integrate neuroimaging data, machine learning (ML) approaches as an area of artificial intelligence (AI) have been used to learn patterns in empirical data through developing computational models in order to make predictions on new data. Using neuroimaging data, ML algorithms as part of the multivariate approaches take into account the inter-correlation between voxels, allowing statistical inferences at a single subject level, and have the potential to aid in making individual diagnostic and prognostic decisions (Quaak et al., 2021). Among all the ML algorithms, deep learning (DL) models are becoming the state-of-the-art algorithms based on the concept of the artificial neural network (ANN) by building multiple interlinked artificial neurons that stimulate the biological functions of the human brain. DL methods are a type of representation-learning methods that can automatically identify the optimal representation from the raw data without needing prior feature selection through a hierarchical structure that involves the application of sequential non-linear transformations to the raw data. Hence, these transformations generate an increasingly higher level of abstraction and less bias-prone patterns of the given data (Vieira et al., 2017; Liu et al., 2022; Yin et al., 2022). A DL model with multiple hidden layers has achieved unprecedented classification performance relative to the conventional ML models in the classification of patients with neurological and psychiatric disorders using neuroimaging, including Alzheimer’s disease (AD) (Hazarika et al., 2023), Parkinson’s disease (PD), mild cognitive impairment (MCI) (Kang et al., 2020), autism spectrum disorder (Wang et al., 2019), major depression disorder (MDD) (Liu et al., 2022), and schizophrenia (Li et al., 2020). However, to date, to our knowledge, no study has been conducted using DL-based approach in the identification of patients with chronic mTBI incorporating multiple functional imaging modalities.

The present study proposed a novel approach employing several rs-fMRI metrics, including fALFF, DC, FCS, ReHo, and VMHC, and fluorodeoxyglucose-PET (FDG-PET) in discrimination of patients with chronic mTBI compared to HCs. An ANN framework was designed by combining autoencoder (AE) and multilayer perceptron (MLP) for binary classification. Furthermore, the most discriminative regions of interest (ROIs) were defined through the first layer of ANN. Finally, rs-fMRI metrics and PET features were fused via a multimodality model. We hypothesized that each single model is able to provide informative diagnostic performance, while the multimodality approach would improve the classification performance. Additionally, the results of the model interpretation indicating the most discriminative ROIs would be consistent with the findings from the literature on mTBI.

## Methods

### Participants

A total of 83 patients, including 31 men (age: 44 ± 14.6 years) and 52 women (age: 49 ± 13.6 years) who were experiencing chronic symptoms due to a mild traumatic brain injury, and 40 matched healthy controls, including 21 men (age: 41 ± 9.4 years) and 19 women (age: 39 ± 10.6 years), participated in this study. mTBI was defined by the Mayo Classification System for Traumatic Brain Injury Severity, in which an injury was classified as mild if there were a loss of momentary consciousness for <30 min and amnesia for <24 h, with no positive MRI findings (Malec et al., 2007). Participants had to report a history of one or more prior TBI (one or multiple) for meeting the criteria for mild concussion (loss of consciousness for <30 min, no significant amnesia, and no structural injury to the brain such as a hematoma, contusion, dura penetration, or brain stem injury). They had to meet ICD-10 criteria for post-concussion syndrome based on symptoms that were the result of the TBI and could include headache, dizziness, irritability, cognitive problems, emotional problems (e.g., depression or anxiety), hypersensitivity to auditory or visual stimuli, balance problems, insomnia, or other subjective complaints specifically associated with the TBI. Patients also had to report that the symptoms lasted for a minimum of 3 months from the last concussion.

Written informed consent, approved by the institutional review board of Thomas Jefferson University, was obtained from all participants, and the study was registered on clinicaltrials.gov with the following identifier: NCT03241732. Participants were recruited from the local community by self-referral and from local neurology offices and were excluded if they had a history of other neurological disorders, significant medical illness, a current substance-use disorder, or current Diagnostic and Statistical Manual of Mental Disorders, 4th Edition (DSM-IV) Axis I psychiatric illness.

For the control group, individuals were excluded if they had a history of previous TBI, a history of other neurological disorders, significant systemic medical illness, a current substance use disorder, and a current Diagnostic and Statistical Manual of Mental Disorders, 4th Edition (DSM-IV) Axis I psychiatric illness (Vedaei et al., 2023).

### Imaging protocol

For each individual, an MRI examination was performed using a 3 T Siemens Biograph mMR Positron Emission Tomography-MR (mMR PET-MR) scanner with a 32-channel head coil. A structural T1-weighted image was acquired to use during the segmentation and registration steps of data preprocessing. The MRI parameters for the anatomical T1-weighted sequence were as follows: repetition time = 1,600 ms, echo time = 2.46 ms, field of view (FOV) = 250 mm × 250 mm, matrix = 512 × 512, voxel size = 0.49 × 0.49, and 176 slices with slice thickness = 1 mm.

In addition, a resting-state BOLD scan was administered using an echo planar imaging (EPI) sequence using the following imaging parameters: FOV = 240 mm × 240 mm; voxel size = 3 mm × 3 mm × 4 mm; TR = 2000.0 ms; TE = 30 ms; slice thickness = 4 mm; number of slices = 34; number of volumes = 180; and acquisition time = 366 s. During rs-fMRI, the participants were asked to close their eyes and rest quietly without thinking about anything.

The PET imaging was performed utilizing general standard-of-care procedures. For the PET scan, an intravenous catheter was placed in the antecubital vein of the arm and 148–296 MBq of FDG was injected via manual bolus over a period of less than 1 min. The intravenous catheter was removed, and the patient was then asked to lie still in a chair in a dimly lit room with minimal ambient environmental stimuli for approximately 30 min to allow for FDG uptake. PET images and MRI images were simultaneously obtained on a 3 T Siemens mMR PET-MRI scanner (Siemens Medical Solutions USA, Inc., Malvern, PA). The PET imaging was a 20 min single FOV, list mode continuous acquisition. All PET/MRI acquisitions included the sequence used for the derivation of standard MR attenuation correction maps based on the Dixon sequence that allows for the separation of water, fat, and bone signals and automatically applies the calculated attenuation correction. Other standard imaging corrections were applied for detector efficiency, decay, dead time, attenuation, and scatter corrections. Image reconstruction used a Gaussian filter set at 2 mm full width at half maximum (FWHM) and was based on an ordinary Poisson ordered-subsets expectation maximization algorithm with 4 iterations and 21 subsets producing an image with a matrix size of 344 × 344 pixels and a voxel size of 1×1×2 mm. All the subjects signed the institutionally approved IRB consent form.

### Data processing and feature selection

#### Resting-state functional MRI processing

For all the participants, rs-fMRI data were preprocessed using Data Processing Assistant for resting-state fMRI (DPABI_V7.0_230110; http://rfmri.org/DPARSF) (Yan et al., 2016) in several steps. Data processing includes discarding the first 10 volumes to remove potential bias in the analysis arising from the initial transient approach to steady-state magnetization and adaptation of participants in the scanning environment; slice timing and motion correction using six rigid body motion parameters, co-registration and normalization of T1-weighted and the mean of realigned EPI images to the EPI template in Montreal Neurological Institute (MNI) space with a resampling voxel size of 3 × 3 × 3 mm; employing the Friston 24-parameter model (the 24 parameters including 6 head motion parameters, 6 head motion parameters of the previous scan, and the 12 corresponding squared items) to regress out the micro head motion effects from the realigned data (Friston et al., 1996); and regressing out the signal from the white matter (WM) and cerebrospinal fluid (CSF) with a temporal band-pass of 0.01–0.08 Hz to reduce the effects of low-frequency drifts and high-frequency respiratory and cardiac noise. The exclusion criteria included excessive head motion (> 2.0 mm translation and/or 2.0^0^ rotation) (Power et al., 2014). The head motion was measured using frame-wise displacement (FD). No participant was excluded due to the exclusion criteria. Furthermore, the mean FDs were not different between the mTBI patient and HC groups (independent *t*-test, *p*-value = 0.2).

#### Fractional amplitude of low frequency fluctuation

After preprocessing for each participant, spatial smoothing with a Gaussian kernel of 6 mm FWHM was applied. fALFF is the fast Fourier transformation (FFT) of rs-fMRI time series and is calculated as the ratio of power in the low-frequency band (0.01–0.08 Hz) to the power of the entire frequency range (0–0.25 Hz) (Zou et al., 2008). Therefore, with FFT, the time courses of the rs-fMRI signal were converted to the frequency domain; then, the voxel-wise fALFF maps were generated as the ratio of power in the low-frequency band (0.01–0.08 Hz) to the power of the entire frequency range (0–0.25 Hz). The normalized derivation of ALFF describes the spontaneous brain activity across the whole brain by measuring the amplitude of neural activity in the low-frequency range (0.01–0.08 Hz) relative to the entire frequency range (e.g., 0–0.25 Hz if TR = 2 s) amplitude. Hence, this frequency range has been recommended to use fALFF rather than ALFF due to its reliability to exclude non-specific signal noises, such as physiological artifacts (Zou et al., 2008). To ensure standardization, the fALFF maps were transformed to z-scores using Fisher’s z-transform, and zfALFF maps were generated (Vedaei et al., 2022, 2023).

#### Functional connectivity strength

For each participant, the FCS maps were measured by estimating the Pearson correlation coefficients between the time series of each voxel and all other voxels in the entire brain (Dai et al., 2015). For a given voxel i , FCS was measured using the following equation (Eq. 1) (Dai et al., 2015):


(1) $$FCS\;\left(i\right)=\frac{1}{N_{\mathrm{voxels}}}\;\overset{N_{\mathrm{voxels}}}{\underset{j\neq i}{\sum }}Z_{ij}\;r_{ij}>r_{0}\text{,}$$

where $z_{ij}$ refers to the Fisher’s Z-transformed version of the correlation coefficient, $r_{ij}$ refers to the values between voxel i and voxel j , and $r_{0}$ refers to a correlation threshold that was used to exclude weak correlations possibly arising from noises ( $r_{0}$ =0.2 in this study). $r_{ij}$ was converted to $Z_{ij}$ using Fisher’s Z-transformation. $N_{\mathrm{voxels}}$ is also defined as the total number of voxels within the gray matter mask (Vedaei et al., 2023).

#### Degree centrality

DC is the graph theory-based metric representing the number of links incident upon each brain voxel as a node. It measures the functional connection between each voxel and the voxels in the entire brain that is defined as an edge. By computing the Pearson correlation coefficient between the time series of each pair of voxels, a correlation matrix can be obtained. To exclude the weak correlations that could be induced by physiological noise, the threshold of 0.25 was used to generate the undirected adjacency matrix. Thus, for each voxel, DC was calculated as the sum of its connections with other voxels. For standardization purposes, the weighted DC was transformed to z-scores using Fisher’s z-transform. Finally, the zDC map was smoothed with an isotropic 6 mm FWHM Gaussian kernel (Wang et al., 2021).

#### Regional homogeneity

ReHo is a voxel-based measure of brain activity that evaluates the similarity or synchronization between the time series of a given brain activity and its nearest neighbors. This synchronization is accomplished on a voxel-based basis by calculating Kendall’s coefficient of concordance (KCC) with a given time series that is assigned as the center voxel and that of its nearest 26 neighboring voxels (Eq. 2) (Zang et al., 2004; Ji et al., 2020),


(2) $$w=\frac{\sum \left(R_{i}\right)-n\;{\left({\bar{R}}_{i}\right)}^{2}}{\frac{1}{12}K^{2}\left(n^{3}-n\right)}\text{.}$$

In this formula, w is the KCC (ranging from 0 to 1) among given voxels; K is the number of neighboring voxels ( K = 26); ${\bar{R}}_{i}$ is the mean rank across nearest neighbors (26 voxels) at the i th time point; and n is the total number of time points. For standardization purposes, the ReHo value at each voxel was transformed to the standardized Fisher’s Z-transformation to obtain the zReHo maps. Spatial smoothing with an isotropic 6 mm FWHM Gaussian kernel was performed after ReHo calculation (Vedaei et al., 2023).

#### Voxel-mirrored homotopic connectivity

VMHC is designed to directly compare the interhemispheric resting-state FC. This process can also measure the correlations between blood oxygen level-dependent (BOLD) time series and reflect the communication pattern of information between two cerebral hemispheres. VMHC measures the synchrony in spontaneous activity between geometrically corresponding interhemispheric regions between pairs of symmetric voxels. It is quantified by calculating the Pearson correlation coefficient with the time series of each voxel and that of its symmetric inter-hemispheric counterpart. After generating the VMHC maps, the correlation values were transformed to z-scores using Fisher’s z-transform to generate zVMHC maps (Zuo et al., 2010).

#### Positron emission tomography processing

The PET data were processed using the PETPVE12 toolbox incorporated as a part of SPM12 running on MATLAB_R2023a (Gonzalez-Escamilla et al., 2017). The processing steps included skull stripping and segmentation of anatomical T1-weighted to the GM, WM, and CSF; co-registration of PET data to the anatomical T1-weighted data using rigid-body transformations; partial volume effect correction (PVEc) of PET data to correct the voxel spillage caused by low-resolution PET scanners using the Müller-Gärtner method (Müller-Gärtner et al., 1992); glucose intensity normalization by computing the standard uptake value ratios (SUVR) using the whole cerebellar signal in the individual raw PET data as the reference signal; normalization of PET data to the MNI template space; and spatial smoothing of PVEc PET data with a 6 mm FWHM Gaussian kernel (Byrnes et al., 2014).

All rs-fMRI and PET data were parcellated into 116 regions of interest (ROIs) according to the Automated Anatomical Labeling (AAL) atlas (Tzourio-Mazoyer et al., 2002); therefore, for each metric, the average of the intensities from 116 ROIs was extracted and used as input features of the ANN algorithm (Zou et al., 2017; Hao et al., 2020).

### Proposed classification method

**Figure 1:**
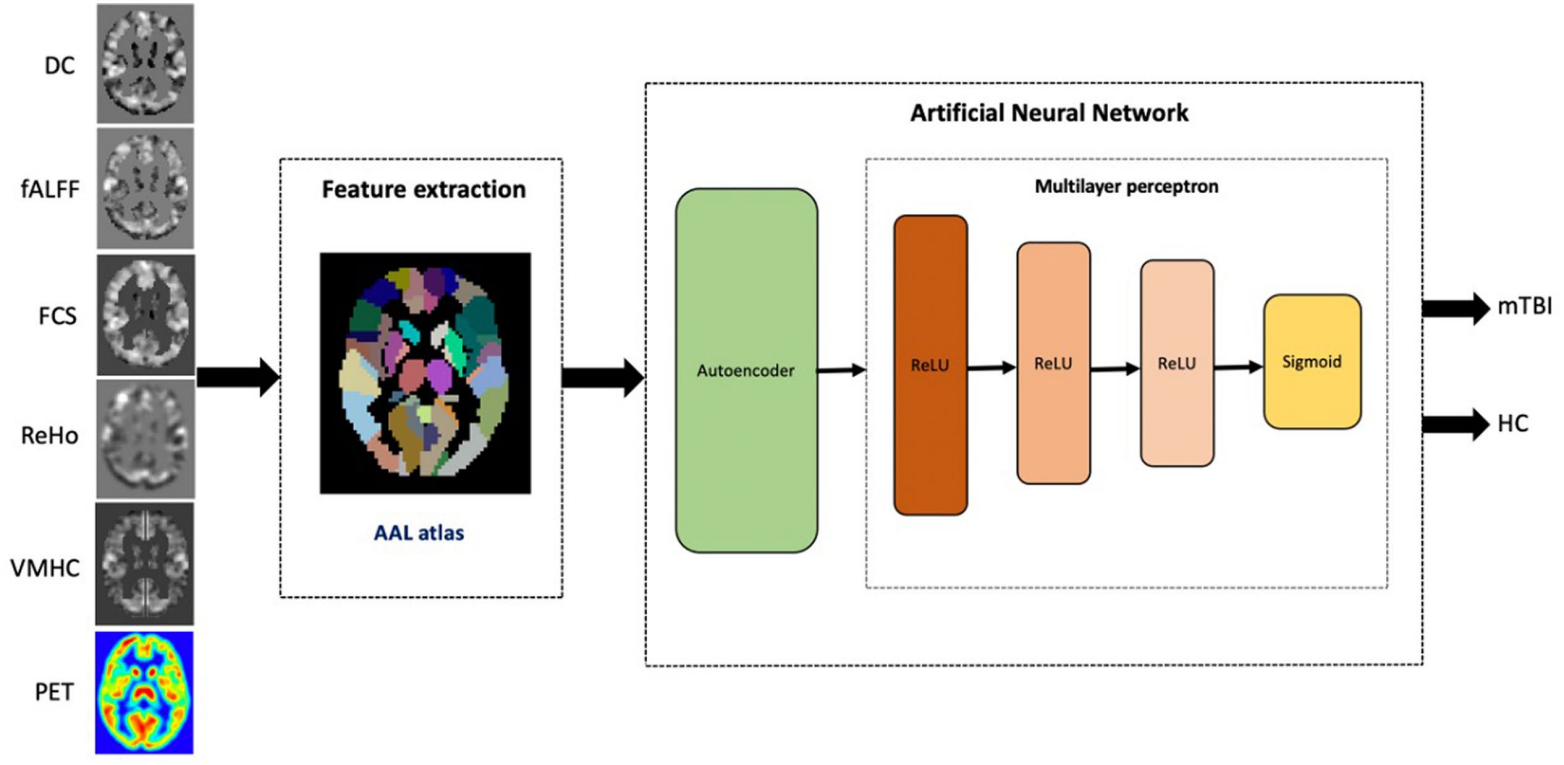
The proposed workflow for single modality mTBI classification based on rs-fMRI metrics and PET data.

Figure 1 illustrates the framework of mTBI versus HCs classification for each single rs-fMRI metric and PET data. Our ANN architecture included multiple levels of abstraction, including extracting the latent representation features using AE, and hidden layers of MLP employing the ReLU function and sigmoid utilized in the output layer for binary classification.

**Figure 2:**
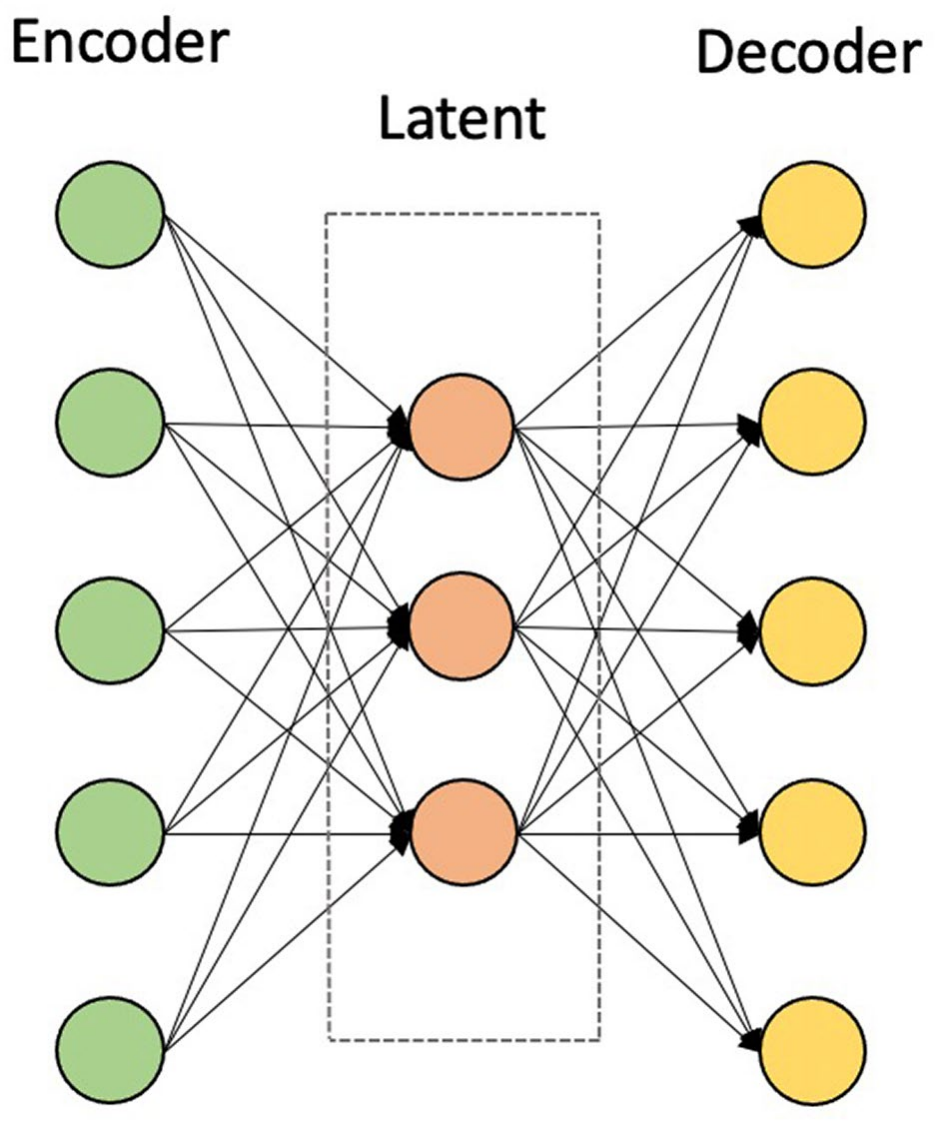
The structure of the autoencoder consisting of an encoder and a decoder.

For the given rs-fMRI metrics and PET data, first, the mean time series of 116 ROIs were extracted as described in the data preprocessing section. For generating training and testing datasets, each dataset was split in a ratio of 80:20 to training and testing datasets. Prior to training the models, each feature in the training dataset was scaled using a MinMaxScaler, which modifies the dataset in a standardized scale within the range between 0 and 1. In addition, the AE was trained, which takes the mean intensities of ROIs as inputs and finds the nonlinear associations among ROIs in an unsupervised manner. AE is used in the pre-training stage by extracting compressed representations from input data by minimizing the reconstruction error. This stage consists of two main modules, namely an encoder and a decoder. The encoder maps the data into a low-dimensional latent representation by learning the useful structure from the input data, while the decoder reconstructs it back to the input data (Figure 2) (Suk et al., 2016; Hao et al., 2020).

After building the AE, the encoder weights were conveyed into an MLP with three hidden layers to adjust the MLP weights and minimize the prediction error into the output layer in a supervised manner. The ReLU activation function ( $f\left(x\right)=\max\;\left(0,x\right))$ was used in the hidden layers of the MLP structure with 62, 32, and 16 nodes, respectively. An MLP is a feed-forward neural network structure that maps input training data to target labels. As such, in our architecture, the weighted sum of all inputs is calculated and then bias is added. Therefore, the result is referred to the subsequent layer, and the activation function is applied. ReLU used in this study has the advantage to alter the non-positive inputs as zero, which act as non-activated nodes. Hence, using ReLU, not all the nodes are active at the same time, which makes it computationally efficient during the training process (Heinsfeld et al., 2018; Thomas et al., 2019).

In the output layer, the sigmoid activation function was executed with a single node to obtain the probability of belonging to the patient group and particularly being used for binary classification. A logistic function maps the input to a value between 0 and 1 interpreted as the probability of the input being labeled to the positive class. The sigmoid function is given in the following equation (Eq. 3):


(3) $$f\left(z\right)=\frac{1}{1+e^{-z}}\text{.}$$

**Figure 3:**
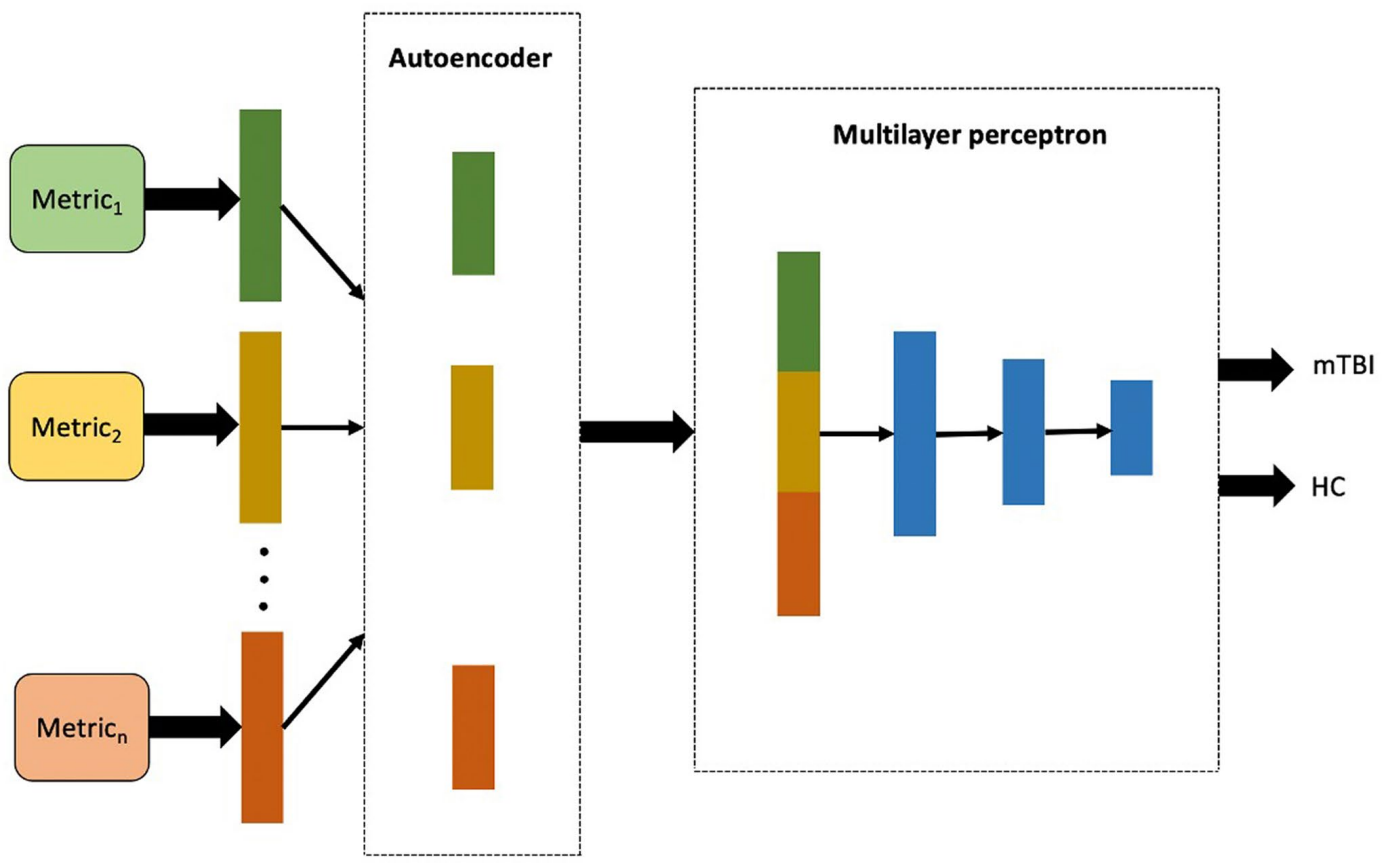
The proposed workflow for multimodality mTBI classification fusing high-level features of each single metric extracted in the first layer of the model.

In the multimodality architecture, the AE-learned features were independently obtained from each metric and then concatenated into a single long vector of input data to the MLP model. The framework of the multimodality architecture is shown in Figure 3. The multimodality model applied for the sets of data included a combination of the whole rs-fMRI metrics as well as adding PET to the rs-fMRI metrics (Zhou et al., 2019).

### Model training and performance

We performed an end-to-end training through a mini-batch size of 1 and 500 epochs to ensure that training was converged at the end (Hazarika et al., 2022). To prevent the model from overfitting, the early stopping technique was employed during training to monitor the performance of the model during each epoch and to terminate the training process when the model performance started to deteriorate (Zhang et al., 2019). The patience parameter was set to five epochs to evaluate the improvement in model performance. Therefore, the best model was saved at the last epoch where an improvement was observed, which is just before the start of the 5-epoch without further improvements. Moreover, the cross-entropy loss function or log loss function was used to estimate the dissimilarity between the predicted probability distribution and the true binary labels. The binary cross-entropy loss function is given in the following equation (Eq. 4):


(4) $$L\left(W,b\right)=\frac{-1}{N}\;\left(\overset{N}{\underset{n=1}{\sum }}y_{n}\;\ln H_{w,b}\;\left(x_{n}\right)+\left(1-y_{n}\right)\;\ln\left(1-H_{w,b}\right)\;\left(x_{n}\right)\right)\text{,}$$

where N is the number of samples; $x_{n}$ and $y_{n}$ are the input and corresponding label of the $n^{th}$ sample; $H_{w,b}$ (.) is the function learned by the neural network; and $H_{w,b}\left(x_{n}\right)$ corresponds to the output of the neural network given the input $x_{n}$ . During the training process, the model aims to minimize the binary cross-entropy loss by adjusting its parameters through the optimization algorithm (Zou et al., 2017). The Adam optimizer with a learning rate of 0.001 was applied to optimize the model’s parameters based on the gradients computed using the cross-entropy loss function (Etminani et al., 2022). In each epoch, through forward propagation, the output value of each layer is calculated; then, the error is propagated through the backpropagation step; and the weight parameters of each layer are adjusted according to the residual error (Hazarika et al., 2022).

To evaluate the results obtained by the ANN, the performance of the models was quantified on the testing dataset via fivefold cross-validation (CV) using the receiver operator characteristic (ROC) curve analysis. The corresponding area under the curve (AUC), accuracy, sensitivity, specificity, F1-score, recall, and precision were obtained (Kingma and Ba, 2015). The equations of the scores are given as follows (Eq. 5–11):


(5) $$AUC=\frac{1}{2}\;\sum_{i=1}^{m-1}\left(x_{i-1}-x_{i}\right)\;\left(y_{i}+y_{i+1}\right)$$

(6) $$\mathrm{Accuracy}=\frac{\left(TP+TN\right)}{\left(TP+FN+TN+FP\right)}$$

(7) $$\mathrm{Sensitivity}=\mathrm{True}\;\mathrm{Positive}\;\mathrm{Rate}=\frac{TP}{\left(TP+FN\right)}$$

(8) $$\mathrm{Specificity}=\mathrm{True}\;\mathrm{Negative}\;\mathrm{Rate}=\frac{TN}{\left(TN+FP\right)}$$

(9) $$\mathrm{Recall}=\frac{TP}{\left(TP+FN\right)}$$

(10) $$\mathrm{Precision}=\frac{TP}{\left(TP+FP\right)}$$

(11) $$F1-\mathrm{score}=\frac{2*\left(\mathrm{Precision}*\mathrm{Recall}\right)}{\left(\mathrm{Precision}*\mathrm{Recall}\right)}\text{,}$$

where $\left\{\left(x_{1},y_{1}\right),\left(x_{2},y_{2}\right),\ldots ,\left(x_{n},y_{n}\right)\right\}$ are the sequential coordinates connection formed the ROC curve. Furthermore, the true positive (TP), false negative (FN), true negative (TN), and false positive (FP) correspond to the number of mTBI correctly classified, the number of mTBI predicted to be HCs, the number of HCs correctly classified, and the number of HCs predicted to be mTBI, respectively.

Given the limited number of samples, CV was used to reuse data five times by dividing each dataset to five parts. In each CV experiment, four of them were used as the training set, and one as a test set. Hence, in each experiment, the scores were measured independently of the test sets, and the average of the scores was recorded for each model (Hazarika et al., 2022).

### The most discriminative features

The most important ROIs were determined as the most frequently selected features in the single modality models, which can potentially be used as imaging biomarkers in the clinical diagnosis of mTBI. For each single metric, the features were ranked based on the weight matrix generated in the first layer of the ANN (AE as encoder). In each fold, the top 10 ROIs for each imaging metric were selected based on the summation of absolute values along the row in the weight matrix for each ROI. Finally, the features with the highest selection frequency among fivefold CV were defined as the top 10 ROIs for single rs-fMRI metrics and PET measurements (Zhou et al., 2019).

## Results

### Demographic characteristics

Demographic statistical analysis showed no significant difference between the age of HCs and the mTBI patient groups (independent *t*-test, *p*-value = 0.07), as well as in the proportion of male and female individuals in the two groups (Chi-squared; χ2 = 2.5, *p*-value = 0.11) (Table 1).

**Table 1:** Demographic characteristics of participants in the mTBI patient and HC groups.

Demographics	HCs (*n* = 40)	mTBI (*n* = 83)	*p*-value	Statistic
Age (year) (SD)	40.32 (9.9)	47.0 (14.02)	0.07^a^	1.8^a^
Sex (M/F)	21: 19	31: 52	0.11^b^	2.5^b^
Injury-to-imaging interval (95% CI) (months)	–	25–38		
Single concussion vs. multiple (single: multiple)	–	32: 51		

HCs, healthy controls; mTBI, mild traumatic brain injury; SD, standard deviation; CI, confidence interval.

a *p*-value and T-statistic obtained by two-sample *t*-test.

b *p*-value and χ2-statistic obtained using Chi-square *t*-test.

### Classification performance

**Figure 4:**
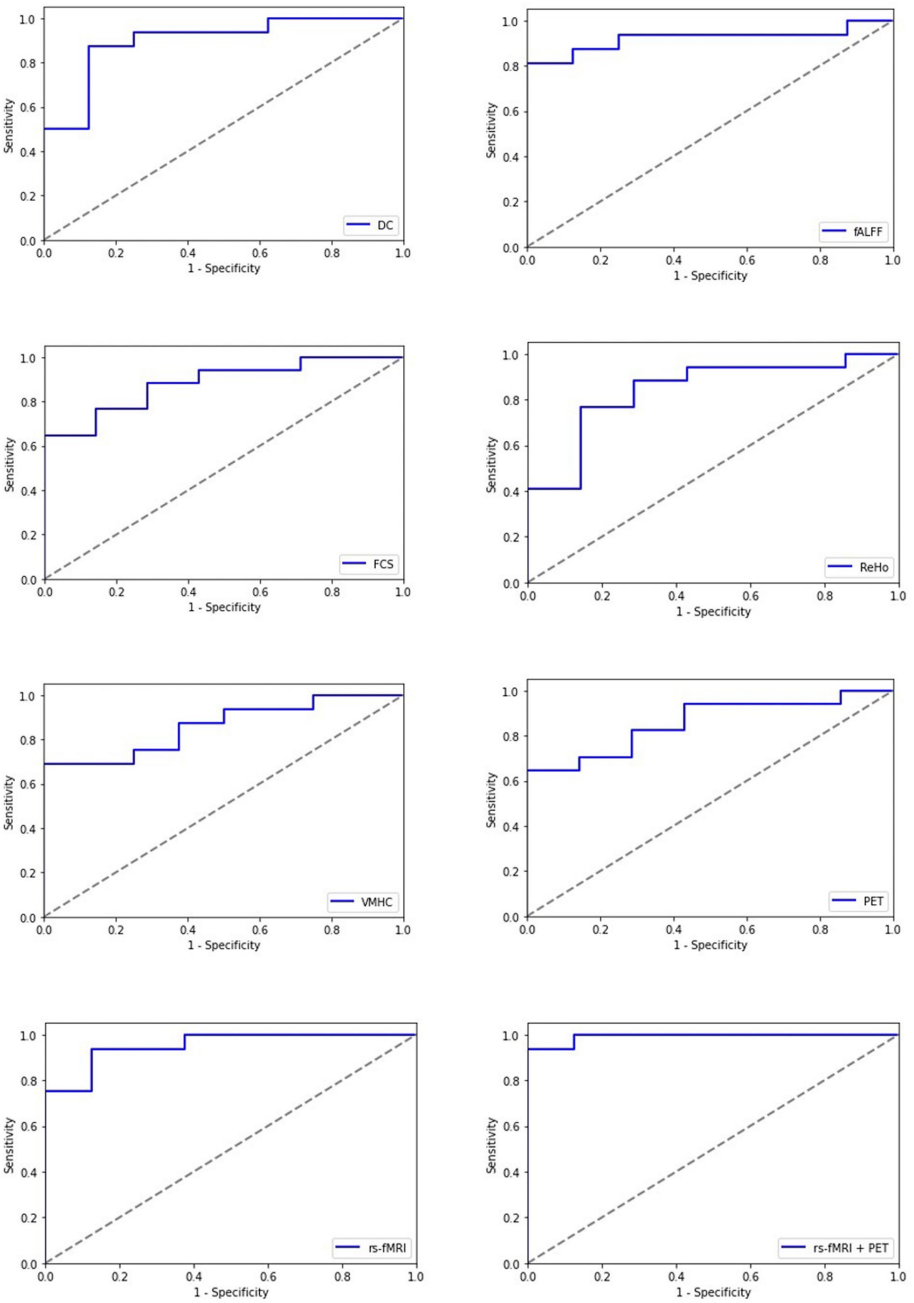
Receiving operating characteristic curve (ROC) of the artificial neural network (ANN) model for each single modality and multimodality metrics of rs-fMRI and PET data; rs-fMRI = DC + fALFF + FCS + ReHo + VMHC; DC, degree centrality; fALFF, fractional amplitude of low-frequency fluctuation; FCS, functional connectivity strength; ReHo, regional homogeneity; VMHC, voxel mirrored homotopic connectivity.

In reporting the classification performance of chronic mTBI patients versus HCs, the percentage value of AUC, accuracy, sensitivity, specificity, recall, precision, and F1-score was obtained on testing datasets for each single modality and multimodality models. The list of the scores for single rs-fMRI metrics, PET, and cognitive biomarkers as well as the multimodality models, including rs-fMRI (DC + fALFF + FCS + ReHo + VMHC) and rs-fMRI + PET (DC + fALFF + FCS + ReHo + VMHC + PET), are provided in Table 2. Moreover, Figure 4 represents the results of the ROC analysis.

**Table 2:** Classification performances of the single metrics of rs-fMRI and PET and multimodality models (mean ± standard deviation).

Features	AUC	Accuracy	Sensitivity	Specificity	F1-score	Precision	Recall
DC	87.95 ± 6.1	91.67 ± 4.0	98.75 ± 0.1	77.14 ± 8.2	94.21 ± 2.8	90.31 ± 5.2	98.75 ± 0.1
fALFF	81.25 ± 2.1	86.67 ± 8.1	95.00 ± 6.8	67.50 ± 2.7	91.01 ± 5.4	88.13 ± 9.4	95.00 ± 6.8
FCS	78.66 ± 7.7	84.17 ± 5.2	93.75 ± 7.2	63.57 ± 1.8	88.94 ± 3.7	85.24 ± 6.6	93.75 ± 7.2
ReHo	75.13 ± 1.5	80.83 ± 7.2	91.32 ± 4.6	58.93 ± 3.3	86.71 ± 5.0	84.42 ± 1.0	91.32 ± 4.6
VMHC	78.21 ± 5.4	85.00 ± 4.2	97.50 ± 8.2	58.93 ± 1.4	89.85 ± 3.6	83.62 ± 5.2	97.50 ± 8.2
PET	72.04 ± 9.6	79.17 ± 5.6	92.65 ± 4.2	51.43 ± 1.9	85.85 ± 3.4	80.49 ± 4.9	92.65 ± 4.2
rs-fMRI	92.32 ± 7.1	94.17 ± 4.7	97.50 ± 6.5	87.14 ± 3.3	95.82 ± 1.7	94.55 ± 6.2	97.50 ± 2.3
rs-fMRI + PET	93.75 ± 3.4	95.83 ± 6.2	100 ± 1.1	87.50 ± 2.1	97.04 ± 1.8	94.38 ± 3.2	100 ± 0.2

AUC, area under the receiver operator curve; DC, degree centrality; fALFF, fractional amplitude of low-frequency fluctuation; FCS, functional connectivity strength; ReHo, regional homogeneity; VMHC, voxel mirrored homotopic connectivity; rs-fMRI, resting-state functional magnetic resonance imaging (rs-fMRI = DC + fALFF + FCS + ReHo + VMHC).

Among imaging modalities, DC showed the highest classification performance. The AUC values for single modality models were 87.95, 81.25, 78.66, 75.13, 78.21, and 72.04% for DC, fALFF, FCS, ReHo, VMHC, and PET, respectively. Moreover, we found that the multimodality models outperform the single modality models with improved classification scores. The AUC values for the rs-fMRI and rs-fMRI + PET models were 92.32 and 93.75%, respectively.

### The most discriminative features

**Figure 5:**
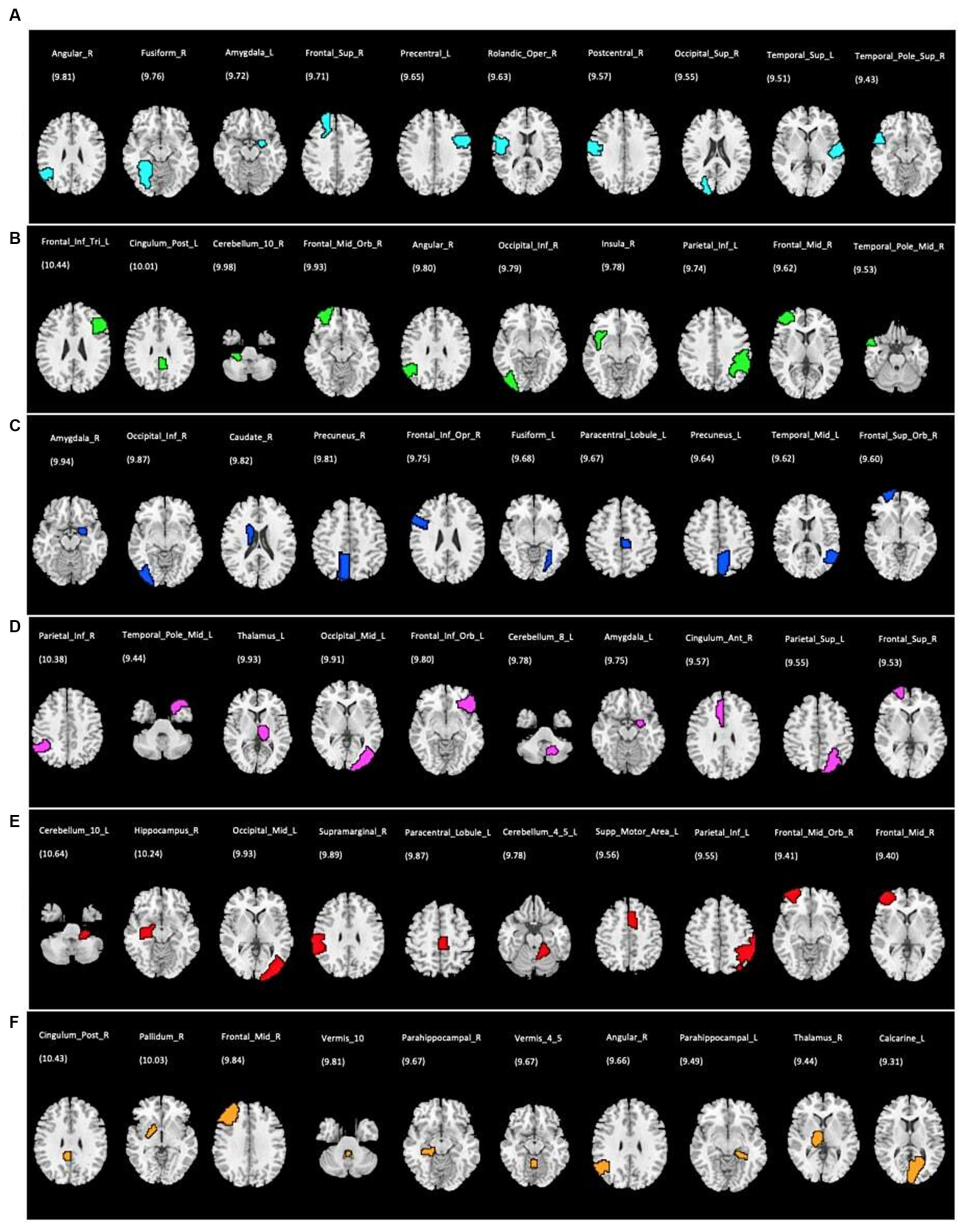
Top 10 ROIs defined as the most discriminative features for each single rs-fMRI metrics and PET data; **(A)** DC, degree centrality; **(B)** fALFF, fractional amplitude of low-frequency fluctuation; **(C)** FCS, functional connectivity strength; **(D)** ReHo, regional homogeneity; **(E)** VMHC, voxel mirrored homotopic connectivity; **(F)** PET, positron emission tomography. The corresponding discriminative weights for each ROI are shown as weight%.

Using single modality models, the top 10 ROIs for each metric and 4 cognitive biomarkers were identified. The list of the 10 ROIs for single metrics is summarized in Table 3 and is shown in Figure 5. Among rs-fMRI metrics, the top ROIs for DC were in the superior temporal, frontal, and occipital cortices, the angular gyrus, the fusiform gyrus, the precentral gyrus, and the postcentral gyrus. For fALFF, top ROIs were in the middle temporal and frontal cortices; the inferior parietal and occipital cortices; the angular gyrus; and the cerebellum insula. Similarly, top ROIs for FCS are located in the middle temporal, superior frontal, and inferior occipital cortices, the precuneus, caudate, amygdala, the fusiform gyrus, and the paracentral gyrus. Moreover, ReHo showed the top ROIs in the middle temporal, occipital, inferior and superior frontal, superior parietal cortices, the amygdala, the thalamus, the cerebellum, and the anterior cingulate gyrus. The top ROIs for VMHC were defined in the middle frontal, occipital, inferior parietal cortices, the hippocampus, the cerebellum, the paracentral gyrus, and the supramarginal gyrus. For PET modality, the top 10 ROIs were in the middle frontal cortex, parahippocampal gyrus, pallidum, thalamus, angular gyrus, posterior cingulate, calcarine, and vermis.

**Table 3:** Top 10 ROIs defined as the most discriminative features among single rs-fMRI metrics and PET data based on the AAL atlas.

fALFF (weight%)	FCS (weight%)	DC (weight%)	ReHo (weight%)	VMHC (weight%)	PET (weight%)
Frontal_Inf_Tri_L (10.44)	Amygdala_R (9.94)	Angular_R (9.81)	Parietal_Inf_R (10.38)	Cerebelum_10_L (10.64)	Cingulum_Post_R (10.43)
Cingulum_Post_L (10.01)	Occipital_Inf_R (9.87)	Fusiform_R (9.76)	Temporal_Pole_ Mid_L (9.44)	Hippocampus_R (10.24)	Pallidum_R (10.03)
Cerebelum_10_R (9.98)	Caudate_R (9.82)	Amygdala_L (9.72)	Thalamus_L (9.93)	Occipital_Mid_L (9.93)	Frontal_Mid_R (9.84)
Frontal_Mid_Orb_R (9.93)	Precuneus_R (9.81)	Frontal_Sup_R (9.71)	Occipital_Mid_ L (9.91)	SupraMarginal_R (9.89)	Vermis_10 (9.81)
Angular_R (9.80)	Frontal_Inf_ Oper_R (9.75)	Precentral_L (9.65)	Frontal_Inf_Orb_ L (9.80)	Paracentral_Lobule_L (9.87)	ParaHippocampal_R (9.67)
Occipital_Inf_R (9.79)	Fusiform_L (6.68)	Rolandic_ Oper_R (9.63)	Cerebelum_8_L (9.78)	Cerebelum_4_5_L (9.78)	Vermis_4_5 (9.67)
Insula_R (9.78)	Paracentral_ Lobule_L (6.67)	Postcentral_R (9.57)	Amygdala_L (9.75)	Supp_Motor_Area_L (9.56)	Angular_R (9.66)
Parietal_Inf_L (9.74)	Precuneus_L (9.64)	Occipital_Sup _R (9.55)	Cingulum_Ant_R (9.57)	Parietal_Inf_L (9.55)	ParaHippocampal_L (9.49)
Frontal_Mid_R (9.62)	Temporal_Mid_L (9.62)	Temporal_Sup_L (9.51)	Parietal_Sup_L (9.55)	Frontal_Mid_Orb_ R (9.41)	Thalamus_R (9.44)
Temporal_Pole_ Mid_R (9.53)	Frontal_Sup_Orb_R (9.60)	Temporal_Pole_ Sup_R (9.43)	Frontal_Sup_R (9.53)	Frontal_Mid_R (9.40)	Calcarine_L (9.31)

ROIs, regions of interest; DC, degree centrality; fALFF, fractional amplitude of low-frequency fluctuation; FCS, functional connectivity strength; ReHo, regional homogeneity; VMHC, voxel mirrored homotopic connectivity; R, right; L, left. The corresponding discriminative weights for each ROI are shown as weight% (Supplementary material S1).

## Discussion

In the need for robust biomarkers to differentiate between chronic mTBI patients and HCs, this study proposed a novel automatic DL-based method utilizing single and multimodal neuroimaging metrics of rs-fMRI and PET. We developed an ANN architecture employing unsupervised feature generation of an AE to extract the latent representations and several layers of MLP to distinguish between patients and HCs using single metrics and a combination of rs-fMRI and PET imaging. In the multimodality model, the potential features of rs-fMRI and PET were extracted independently and combined into the hierarchical architecture. Considering the complicated pathology of mTBI involving brain function alteration, it could be challenging to diagnose the patients at a chronic stage by relying upon conventional patient screening and structural imaging alone. Moreover, each single imaging modality could capture part of the neuropathology information related to the brain alteration, and hence, a combination of them may provide complementary information for the classification of patients from normal cases. Our findings illustrated that, although each single imaging modality provides robust classification performance, integration of multiple neuroimaging data in the multimodality model enhances the classification accuracy and is superior to the other state-of-the-art methods.

In recent years, several studies have attempted to develop automatic DL-based classification methods incorporating neuroimaging data to distinguish between patients with neurological and psychiatric disorders and normal subjects. While the body of literature on AD, MCI, PD, schizophrenia, MDD, and autism is increasingly growing, only a few studies have applied ML algorithms, including ANN, to other neuropsychiatric disorders including mTBI. Resting-state fMRI and PET data have been used most often in DL models achieving high classification accuracies. Xing et al. used rs-fMRI data with an FC matrix of 90 ROIs from the AAL atlas as the input features of the DL architecture using a convolutional neural network (CNN) and reported an accuracy of 66.88% for the identification of patients with autism from among HCs (Ebrahimighahnavieh et al., 2020). Moreover, a recent study developed a deep neural network (DNN) model to discriminate patients with PD from HCs using rs-fMRI data. They found the highest accuracy of 96.4% by employing the DNN binary method, which was superior compared to the conventional ML algorithms that achieved 91.4 and 83.7% accuracy using gradient boost and support vector machine (SVM), respectively (Xing et al., 2019). Moreover, a prior study developed a DL model using the CNN architecture to classify subjects with sleep behavior disorder (SBD) with and without MCI using FDG-PET data and reported a classification accuracy of 70% (Xu et al., 2023). Similar to our study, Zou et al. incorporated several rs-fMRI metrics including fALFF, ReHo, and VMHC into a 3D CNN model to differentiate subjects with attention deficit hyperactivity disorder (ADHD) from normal subjects and reported a mean classification accuracy of 66.04 and 69.15% for single modality and multimodality 3D CNN, respectively (Zou et al., 2017). Similarly, Ghanbari et al. employed single and combined rs-fMRI metrics including fALFF, ReHo, and VMHC in a classification model of 3D CNN to identify patients with schizophrenia from HCs and found the accuracy of 72.20, 79.55, 87.63, and 90.91% for fALFF, ReHo, VMHC, and multimodality models, respectively (Ryoo et al., 2022). While many studies have been carried out on the classification between the patient and control groups using conventional ML algorithms, some recent studies have shown the capability and superiority of DNN with several hidden layers to extract lower-to-higher level information through several hidden layers. In the classification of patients with schizophrenia using rs-fMRI measures compared SVM and DNN models, Kim et al. reported a lower error rate and better performance of the DNN model compared to SVM. They also incorporated AE in the pre-training step to minimize the reconstruction error of the input sample and showed that the average error rate is lower with the pre-training AE layer and a minimum of 2–3 hidden layers in the DNN. Finally, they concluded that these approaches using DNN architectures can be useful in developing diagnostic tools for other neuropsychological disorders (Kim et al., 2016). Similar to our study, a prior study incorporated stacked AE in the neural network architecture to extract the high-level features of the FC pattern among 116 ROIs of the AAL atlas and finally passed them through the softmax function to classify patients with autism from normal cases (Wang et al., 2019).

A number of recent studies proposed enhancement of ML classification models employing combined data compared to single modality models (Kim et al., 2016; Kang et al., 2020; Ghanbari et al., 2023). Zhou et al. developed a DL classification model using stacked AE and MLP to distinguish between patients with AD and MCI and HCs by incorporating structural MRI and PET imaging (from Alzheimer’s disease neuroimaging initiative (ADNI) dataset), as well as genetic data. They showed that each single imaging modality provides good classification performance. However, combining multiple imaging modalities and genetic data could improve the accuracy of the classification model (Zhou et al., 2019). Additionally, a previous study in the identification of patients with AD from among HCs executed a DL model using structural MRI, PET (from the ADNI dataset), and neuropsychological diagnosis and suggested that single-modality neuroimaging contains partial information about the brain alteration. However, the multimodal method may provide complementary data associated with the patient’s pathology and enhance the classification performance by achieving robust diagnostic efficacy in discrimination of patients with AD and those with MCI (Zhang et al., 2019). Our study extended the findings of the literature, proposing that the classification performance can be improved by combining multi-level neuroimaging data. We showed that each single rs-fMRI metric and PET imaging provides robust classification accuracy and combines neuroimaging modalities that can enhance the robustness of the classification model. Our results demonstrated the highest classification accuracy of 93.75% for the multimodality model combining rs-fMRI metrics and PET. Furthermore, our study confirmed the findings of the previous studies on the superiority of the DL-based methods compared to conventional ML algorithms without the need for prior engineering data in feature reduction.

To resolve the black box nature of the ANN model and to identify the most discriminative features, the weight matrix generated through the encoding step in the first layer of ANN was utilized. Each row in the weight matrix corresponds to one ROI (for each imaging metric). The top 10 ROIs (for rs-fMRI metrics and PET data) were defined corresponding to the largest absolute values along the rows of the weight matrix. The top ROIs were widespread and not restricted to specific brain areas and networks across the whole imaging data. The most common discriminative brain areas among the neuroimaging metrics were located in the frontal, temporal, parietal, and occipital cortices; the cerebellum; and the sensorimotor and limbic systems.

A number of brain imaging studies have demonstrated the vulnerability of the frontal and temporal lobes in moderate-to-severe brain injury. Particularly, frontal regions have been shown to be involved in pain processing, including headache associated with brain injury (Lu et al., 2018). In addition, neural activity in the temporal region is associated with semantic processing, including verbal memory, language, and executive functioning (Zhang and Shi, 2020). Moreover, the occipital gyrus at the center of the visual cortex has been shown to be at risk of contusion in moderate-to-severe TBI. fALFF and ReHo have been commonly used to analyze rs-fMRI data investigating the pathophysiological aspects of neuropsychiatric disorders. Several studies have shown the alteration of fALFF/ALFF in the middle frontal, temporal, and occipital lobes. These studies reported that spontaneous neural activity alteration might be linked with cognitive impairment, memory, and motor perception disturbances in mTBI patients. Indeed, higher fALFF and brain metabolism in the cortical areas could reflect the potential compensatory response to the damage in patients suffering from an injury (Zhan et al., 2016; Konstantinou et al., 2018; Vedaei et al., 2021).

The majority of mTBI studies have consistently reported the alteration of brain function in the default mode network (DMN) following brain injury. A recent study found increased ReHo in the anterior portion of the DMN in adults with sport-related concussion (Stephenson et al., 2020). Identical to this result, a previous study showed altered ReHo in the frontal, temporal, and parietal lobes, limbic regions including the insula correlated with reduced ability to perform executive functions in acute mTBI patients (Meier et al., 2020). Our findings are in line with the literature demonstrating the involvement of the main brain networks including DMN following mTBI. The parietal cortex has been also identified as one of the top ROIs based on our results. Angular gyrus as the main component of the DMN and parietal cortex has been shown to be linked with cognition, visual word forms, and memories (Vedaei et al., 2021). A prior study revealed an alteration of ReHo in the right angular gyrus in patients with post-traumatic stress disorder (PTSD) compared to normal subjects associated with symptom severity of PTSD (Zhan et al., 2015).

Cerebellum has also been selected among the top discriminative ROIs. The cerebellum has been known to be involved in executive control and regulating behavior through the interconnection with the basal ganglia and cerebral cortex (Fu et al., 2019). In a study on patients with mTBI, Shi et al. showed the abnormality in the cerebellar-temporal connectivity that was linked with motor dysfunction and sensory perception in patients after injury (Baillieux et al., 2010). The basal ganglia are a group of brain structures linked together, with the main part being the limbic system, and have been known to modulate cortical–subcortical emotional responses and are susceptible to impairment following mTBI (Pierce and Péron, 2020; Shi et al., 2021). Consistent with this finding, a previous study revealed the reduction of FC between the insula and temporal and frontal lobes in patients with acute mTBI, as well as its positive correlation with the Montreal Cognitive Assessment (MoCA) test, including orientation and abstraction scores (Bruijel et al., 2022). Furthermore, connectivity within the cingulate-pallidostriatal-thalamic-amygdala pathway has been shown to be linked with a top-down mood-regulation circuit. A recent study found a reduction in rs-fMRI FC between the frontal regions and limbic regions, including the amygdala and thalamus, in mTBI patients compared to normal subjects, which was correlated with depressive symptoms in the patients (Lu et al., 2020).

Our results are in line with the previous literature demonstrating the communication disruption between several brain networks in mTBI patients that might interfere with multiple integrative roles, including mood regulation, motor control, memory, social cognition, and language. A previous study used independent component analysis (ICA) comparing rs-fMRI measures in patients with mTBI and HCs. The results showed alteration in FC between the brain networks, including DMN, limbic system, motor, and visual networks, that could be interpreted as the compensatory mechanism in response to an injury linked with cognitive dysfunction (Luo et al., 2021). Moreover, Schwedt et al. in a study on subjects with post-traumatic headache showed the involvement of the frontal cortex, precuneus, and supramarginal gyrus in pain processing in headache disorders (Stevens et al., 2012). The supramarginal gyrus is a part of the inferior parietal cortex and has been shown to be involved in highly integrated tasks, such as motor attention, verbal working memory, language, and cognitive evaluation of pain, including pain empathy (Schwedt et al., 2017).

An increasing number of studies have reported the engagement of various DMN areas in TBI, including the precuneus, posterior cingulate, ventral anterior cingulate, and medial prefrontal cortex. All these studies suggested that the alteration of brain function in these regions might be correlating with self-awareness, visuospatial awareness, consciousness, and post-concussive complaint severity in these patients (Zhou et al., 2012; Deschamps et al., 2014; Palacios et al., 2017; Lu et al., 2020). As such, interactions between multiple brain regions in different brain networks that are known to exist in mTBI may seem reasonable.

In addition to the DMN, other prominent brain networks have been reported to be affected by TBI including the sensorimotor network (SMN). The postcentral gyrus and paracentral lobule as parts of the SMN are among the top ROIs based on our findings. Motor system dysfunction has been implicated in several studies in patients suffering from headache following TBI, reflecting neural mechanisms in response to pain stimuli (Lu et al., 2018). The SMN is involved in sensory processing, motor learning, movement planning, and functional activity alteration. The SMN could be correlated with the severity of post-concussion symptoms (Amir et al., 2021). Interestingly, it has been proposed that higher executive functioning is positively associated with connectivity between the DMN and SMN and negatively associated with connectivity between the SMN and dorsal attention networks (Reineberg et al., 2018). In general, the findings from FDG-PET imaging studies in mTBI patients are qualitatively consistent with the results of rs-fMRI studies, demonstrating that any alterations of glucose metabolism in several brain regions might be characterized as vulnerable after brain injury, particularly in the DMN, SMN, frontal and temporal lobes, cerebellum, and limbic system (Provenzano et al., 2010; Zhang et al., 2010; Byrnes et al., 2014).

Taken collectively, our results are in agreement with the literature confirming the role of multiple brain hubs and networks in cognitive worsening after brain injury. We speculate that abnormality in the brain function in specific areas measured by neuroimaging tools might be linked with behavioral and executive dysfunction in mTBI patients. Considering the complicated pathologic process of mTBI, multiple neuroimaging metrics may provide complementary information and can be investigated via multimodality classification models. Our results confirm the findings from the literature suggesting that fusion of multiple modalities can produce more powerful classifiers than a single modality. Indeed, the pathological changes across the same ROIs could be investigated using multimodalities, which simultaneously eliminates potential noises in the individual modality features. However, the high classification performance of each single modality proves the effectiveness of one imaging modality in the diagnosis of mTBI when just one or a few of them are available. Moreover, these findings confirm the distribution of the common vulnerable ROIs across the entire brain selected by each single imaging classifier. As such, this experiment may represent the consistency of the outcomes when individual rs-fMRI metrics and PET are utilized (Hao et al., 2016; Li et al., 2019; Hao et al., 2020). Additionally, given the enhancement of the classification performance of both multimodality models (rs-fMRI and rs-fMRI + PET), we speculate that the multimodality of rs-fMRI metrics solely might be used in clinical practice in the diagnosis of mTBI without the need to combine them with PET, particularly due to the cost of nuclear imaging.

Despite the extensive application of DL architectures on neuroimaging data and promising results, the clinical application of DL aiding in the prediction of brain diseases is still in its early stage. Given the need to address the lack of robust biomarkers to identify patients at the individual level, our study proposed a novel automatic DL method promising to assist in the diagnosis of patients. This approach can be extended to more diverse datasets of brain disorders and is used as a supplementary software to MRI and PET systems, improving the prediction strategy of neurodegenerative and psychiatric disorders, particularly in circumstances where there is a missing presence of physicians.

Several limitations need to be considered in the interpretation of this study. First, given the small sample size, there was the risk of overfitting, thereby affecting the generalization of our finding. Future studies need to validate our results with larger datasets from two classes. Second, the rs-fMRI scan length was relatively small. While more recent studies have indicated that longer scans provide better reproducibility, we used a 6 min acquisition to maintain consistent parameters with our prior studies. However, several studies showed that a minimum scan length of 5–7 min is needed for data stability (Van Dijk et al., 2010; Birn et al., 2013). Furthermore, the goal of this study was primarily to assess the ability of the DL models in the classification of mTBIs. As such, future studies may investigate the optimal acquisition times and parameters for the use of DL techniques.

Moreover, the proposed algorithm might be applied to different patient cohorts, thereby assuring the generalization of this approach in the classification of other brain disorders. Finally, given the novelty of the application of ML and the DL models in developing diagnostic tools in mTBI, further studies are needed to incorporate more complicated algorithms and architectures and compare their performance to obtain optimized classifiers that can be used in the diagnosis of mTBIs.

## Conclusion

The present study provided a comprehensive approach to developing an automatic classification tool to classify patients with chronic mTBI utilizing DL-based algorithms. We made single and multimodality classification architectures employing rs-fMRI and PET imaging. Our findings showed relatively high classification performance using single neuroimaging metrics. However, the multimodality model integrating multiple rs-fMRI and PET measurements achieved improved accuracy. These results suggest that DNN classifiers might be extended to quantitative imaging biomarkers providing a new avenue for the prediction of individual patients in the clinical settings.

## Data availability statement

The original contributions presented in the study are included in the article/Supplementary materials, further inquiries can be directed to the corresponding author/s.

## Ethics statement

The studies involving humans were approved by Thomas Jefferson University IRB. The studies were conducted in accordance with the local legislation and institutional requirements. The participants provided their written informed consent to participate in this study. Written informed consent was obtained from the individual(s) for the publication of any potentially identifiable images or data included in this article.

## Author contributions

FV: Conceptualization, Formal analysis, Methodology, Project administration, Writing – original draft. NM: Formal analysis, Methodology, Writing – review & editing. MA: Formal analysis, Methodology, Writing – review & editing. GZ: Formal analysis, Methodology, Writing – review & editing. DM: Conceptualization, Funding acquisition, Resources, Writing – review & editing. NW: Formal analysis, Supervision, Writing – review & editing. EN: Data curation, Writing – review & editing. CH: Data curation, Writing – review & editing. AN: Conceptualization, Investigation, Resources, Supervision, Validation, Writing – review & editing. FM: Conceptualization, Investigation, Methodology, Supervision, Validation, Writing – review & editing.
