# Supplementary material for: Deep learning-based multimodality classification of chronic mild traumatic brain injury using resting-state functional MRI and PET imaging
Source: Front Neurosci. 2024 Jan 19;17:1333725. doi: 10.3389/fnins.2023.1333725 (PMC10837852; doi:10.3389/fnins.2023.1333725)
Supplement: Supplementary file 1 [file Table_1.DOCX]

Supplementary 1. The list of ROIs in the AAL116 atlas:

1. Precentral_L
2. Precentral_R
3. Frontal_Sup_L
4. Frontal_Sup_R
5. Frontal_Sup_Orb_L
6. Frontal_Sup_Orb_R
7. Frontal_Mid_L
8. Frontal_Mid_R
9. Frontal_Mid_Orb_L'
10. Frontal_Mid_Orb_LR
11. Frontal_Inf_Oper_L
12. Frontal_Inf_Oper_R
13. Frontal_Inf_Tri_L
14. Frontal_Inf_Tri_R
15. Frontal_Inf_Orb_L
16. Frontal_Inf_Orb_R
17. Rolandic_Oper_L
18. Rolandic_Oper_R
19. Supp_Motor_Area_L
20. Supp_Motor_Area_R
21. Olfactory_L
22. Olfactory_R
23. Frontal_Sup_Medial_L
24. Frontal_Sup_Medial_R
25. Frontal_Med_Orb_L
26. Frontal_Med_Orb_R
27. Rectus_L
28. Rectus_R
29. Insula_L
30. Insula_R
31. Cingulum_Ant_L
32. Cingulum_Ant_R
33. Cingulum_Mid_L
34. Cingulum_Mid_R
35. Cingulum_Post_L
36. Cingulum_Post_R
37. Hippocampus_L
38. Hippocampus_R
39. ParaHippocampal_L
40. ParaHippocampal_R
41. Amygdala_L
42. Amygdala_R
43. Calcarine_L
44. Calcarine_R
45. Cuneus_L
46. Cuneus_R
47. Lingual_L
48. Lingual_R
49. Occipital_Sup_L
50. Occipital_Sup_R
51. Occipital_Mid_L
52. Occipital_Mid_R
53. Occipital_Inf_L
54. Occipital_Inf_R
55. Fusiform_L
56. Fusiform_R
57. Postcentral_L
58. Postcentral_R
59. Parietal_Sup_L
60. Parietal_Sup_R
61. Parietal_Inf_L
62. Parietal_Inf_R
63. SupraMarginal_L
64. SupraMarginal_R
65. Angular_L
66. Angular_R
67. Precuneus_L
68. Precuneus_R
69. Paracentral_Lobule_L
70. Paracentral_Lobule_R
71. Caudate_L
72. Caudate_R
73. Putamen_L
74. Putamen_R
75. Pallidum_L
76. Pallidum_R
77. Thalamus_L
78. Thalamus_R
79. Heschl_L
80. Heschl_R
81. Temporal_Sup_L
82. Temporal_Sup_R
83. Temporal_Pole_Sup_L
84. Temporal_Pole_Sup_R
85. Temporal_Mid_L
86. Temporal_Mid_R
87. Temporal_Pole_Mid_L
88. Temporal_Pole_Mid_R
89. Temporal_Inf_L
90. Temporal_Inf_R
91. Cerebelum_Crus1_L
92. Cerebelum_Crus1_R
93. Cerebelum_Crus2_L
94. Cerebelum_Crus2_R
95. Cerebelum_3_L
96. Cerebelum_3_R
97. Cerebelum_4_5_L
98. Cerebelum_4_5_R
99. Cerebelum_6_L
100. Cerebelum_6_R
101. Cerebelum_7b_L
102. Cerebelum_7b_R
103. Cerebelum_8_L
104. Cerebelum_8_R
105. Cerebelum_9_L
106. Cerebelum_9_R
107. Cerebelum_10_L
108. Cerebelum_10_R
109. Vermis_1_2
110. Vermis_3
111. Vermis_4_5
112. Vermis_6
113. Vermis_7
114. Vermis_8
115. Vermis_9
116. Vermis_10
